# GLP-1 receptor agonists synergistic effects of metabolic reprogramming and cardioprotection

**DOI:** 10.3389/fendo.2025.1614726

**Published:** 2025-10-13

**Authors:** Xuan Wang, Mengmeng Qi, Lili Yang, Libo Yang, Xiaoyue Wang, Fang Zhang, Yukun Cui, Dongxin Wang, Yangang Wang, Wenshan Lv

**Affiliations:** ^1^ Department of Endocrinology and Metabolism, Affiliated Hospital of Qingdao University, Qingdao, Shandong, China; ^2^ Department of Endocrinology, The Affiliated Taian City Central Hospital of Qingdao University, Tai’an, Shandong, China

**Keywords:** metabolic reprogramming, glucagon-like peptide-1 receptor agonists, heart failure, cardiovascular system, diabetes

## Abstract

Diabetes mellitus, a condition that significantly elevates the incidence and mortality risks associated with cardiovascular diseases, exacerbates the disease burden in China. Glucagon-like peptide-1 receptor agonists (GLP-1RAs) have garnered considerable attention, as they not only regulate blood glucose but also play a vital role in safeguarding the cardiovascular system. Recent research shows that metabolic reprogramming is a key mechanism for the cardioprotective effects of GLP-1RAs. GLP-1RAs can achieve metabolic reprogramming by regulating fatty acid, glucose, and ketone body metabolism, as well as mitochondrial function. This process optimizes cardiac energy metabolism, alleviates oxidative stress, and reduces the risk of cardiovascular diseases. This review provides a comprehensive summary of the energy metabolism under normal cardiac conditions and the metabolic reprogramming involved in diabetes-related heart disease. The potential applications and challenges of targeted metabolic reprogramming in the cardioprotective effects of GLP-1RAs are further discussed.

## 1 Introduction

With the advancement of the social economy and alterations in lifestyle patterns, chronic metabolic disorders like obesity and diabetes have emerged as major public health issues (1, 2). The International Diabetes Federation (IDF) data shows that by 2021, approximately 537 million adults worldwide suffered from diabetes, accounting for nearly one-tenth of the global adult population (3). In China, the number of diabetes patients exceeds 140 million, and it is projected to reach 174 million by 2045 (3). These patients commonly exhibit one or multiple components of metabolic syndrome. Such components are closely linked to a high incidence of cardiovascular disease (CVD) and heart failure (HF), resulting in elevated mortality rates and soaring healthcare costs (4, 5).

Type 2 diabetes mellitus (T2DM) is typically characterized by a gradual decline in β-cell function, often rooted in insulin resistance (6). Currently, the management of diabetes primarily relies on lifestyle modifications and pharmacological interventions to maintain optimal blood glucose levels, thereby preventing or delaying the onset of diabetes-related complications (7, 8).

Metabolic reprogramming represents the process wherein an organism modifies its energy metabolism pathways, such as transitioning from glucose metabolism to fatty acid oxidation or other metabolic routes, in response to environmental alterations or disease conditions. This adjustment is to meet new physiological needs (9). Emerging research has indicated that metabolic reprogramming serves as one of the key mechanisms through which GLP-1RAs confer their cardioprotective effects (10, 11). In the context of GLP-1RAs, metabolic reprogramming involves modulating energy metabolism and regulating cardiac function and pathological remodeling through various mechanisms (12, 13). This strategic approach helps prevent or slow the development of cardiovascular complications associated with diabetes. These effects may further support the cardio protection conferred by GLP-1RAs, a property that has been increasingly recognized in recent decades.

In this review, we will summarize the energy metabolism under normal cardiac conditions and the metabolic reprogramming involved in diabetes-related heart disease. Distinguished from prior reviews, we focus specifically on elucidating the role of GLP-1RAs in modulating cardiac metabolism and function through the lens of metabolic reprogramming. We explore the novel mechanisms by which GLP-1RAs confer cardiovascular protection in diabetic patients, offering fresh insights into their therapeutic potential. Our goal is to provide a broader and more nuanced perspective for future research on GLP-1RA-based therapeutics, potentially paving the way for innovative approaches to managing cardiometabolic diseases.

## 2 Clinical evidence for cardioprotective effects of GLP-1RAs

In the process of literature screening for this study, strict inclusion criteria were followed to ensure research quality and relevance to the research theme: In terms of study type, priority was given to randomized controlled trials (RCTs), prospective cohort studies, systematic reviews. In terms of study subjects, the focus was on patients with T2DM, T2DM patients complicated with cardiovascular diseases, or animal models of diabetes-related heart diseases. Moreover, in terms of outcome indicators, the selection centered on "cardioprotection". Cardiovascular-related indicators included the incidence of major adverse cardiovascular events (MACE), cardiac function indicators, and the progression of atherosclerotic lesions.

A search on PubMed with the keywords "Cardiovascular" and "GLP-1 receptor agonists" for the five-year period spanning from 2020 to 2025 retrieved 2,712 items. Existing evidence indicates that GLP-1RAs have demonstrated remarkable cardioprotective effects (14–19). Cardiovascular outcome trials have indicated that GLP-1RAs can decrease the primary composite outcome of first-time major adverse cardiovascular events (MACE) among diabetes patients (20). A systematic review and meta-analysis of randomized controlled trials demonstrated that GLP-1RAs were linked to a significant decline in the MACE incidence(hazard ratio [HR]: 0.86; 95% confidence interval [CI]: 0.79-0.94; I.: 0% (21). Since 2016, numerous cardiovascular outcome studies have revealed that GLP-1RAs can efficiently prevent cardiovascular events such as acute myocardial infarction and stroke, and also lower related mortality (22). Consequently, current guidelines suggest the use of GLP-1RAs for patients with a history of atherosclerotic vascular disease (23). Preclinical research has shown that GLP-1RAs can decelerate the development and progression of atherosclerotic lesions by stabilizing and reducing plaque vulnerability (24). GLP-1RAs have also been beneficial in both heart failure with reduced ejection fraction (HFrEF) and heart failure with preserved ejection fraction (HFpEF) (25). For instance, a prospective trial reported that semaglutide alleviated heart-failure-related symptoms and physical limitations in HFpEF patients (26). However, more research is required to determine whether semaglutide can reduce clinical heart -failure events in this patient group (27).

Clinical trials have shown that GLP-1RAs have a variety of effects, which are presented in **Table 1**. For example, liraglutide might reduce the occurrence rate of myocardial infarction in high-risk T2DM patients and enhance the clinical outcomes of myocardial infarction (28). Regarding heart failure, liraglutide can remarkably improve the left-ventricular diastolic function, indicating its potential in the treatment of T2DM (29). Tirzepatide, a dual GLP-1/GIP receptor agonist that has been approved for controlling blood sugar in T2DM, has emerging evidence suggesting that it is superior to GLP-1RAs in terms of glycemic control and weight loss (30, 31). In the SURPASS-4 trial, tirzepatide significantly decreased blood pressure, body weight, and HbA1c, and its dual-receptor agonism improved lipid profiles, increased insulin secretion, reduced inflammation, and promoted endothelial integrity (32). A recent randomized and double-blind trial revealed that, for adults with poorly controlled T2DM, oral semaglutide at total doses of 25 mg and 50 mg was more effective than the 14-mg total dose in reducing HbA1c and body weight (33). Nevertheless, whether the cardioprotective effect of GLP-1RAs follows a similar dose-dependent pattern still requires further investigation. This is because the primary and key secondary endpoints of this trial were limited to glycemic control and body weight reduction, with no assessment of cardiovascular outcomes that are used to define cardioprotection. Second, long-term data on the cardioprotective efficacy of high-dose oral semaglutide remain lacking.

**Table 1:** Large-scale clinical trials of GLP-1RAs for the incidence of adverse cardiorenal outcomes.

Kind	Disease type	Study name	Study design	Primary outcomes	MACE HR (95% CI)	Other important findings
Liraglutide	T2DM	LEADER	Randomized, placebo-controlled	Significant reduction in MACE (HR 0.87, 95% CI 0.78-0.97)	HR 0.87 (95% CI 0.78-0.97)	Reduced cardiovascular mortality Marso SP et al. N Engl J Med. 2016
Exenatide	T2DM	EXSCEL	Randomized, placebo-controlled	No significant difference in MACE	HR 0.91 (95% CI 0.82-1.01)	Reduced all-cause mortality
Albiglutide	T2DM	Harmony Outcomes	Randomized, placebo-controlled	Significant reduction in MACE (HR 0.87, 95% CI 0.78-0.97)	HR 0.87 (95% CI 0.78-0.97)	Reduced non-fatal myocardial infarction
Dulaglutide	T2DM	REWIND	Randomized, placebo-controlled	Significant reduction in MACE (HR 0.87, 95% CI 0.78-0.97)	HR 0.87 (95% CI 0.78-0.97)	Reduced non-fatal stroke
Semaglutide	T2DM	SUSTAIN-6	Randomized, placebo-controlled	Significant reduction in MACE (HR 0.74, 95% CI 0.58-0.95)	HR 0.74 (95% CI 0.58-0.95)	Reduced non-fatal stroke
Tirzepatide	T2DM	Meta-analysis	Pooled data from 7 trials	No significant difference in MACE-4 eventsHR 0.80 (95% CI 0.57-1.11)	HR 0.80 (95% CI 0.57-1.11)	Consistent cardiovascular safety across trials

In summary, GLP-1RAs have significant cardioprotective effects in HFrEF, HFpEF and other clinical settings. Their benefits go beyond glycemic control, suggesting potential as a key treatment for cardiovascular diseases. Future research should focus on clarifying their cardioprotective mechanisms and exploring applications in other cardiovascular conditions.

## 3 Normal cardiac energy metabolism and abnormal cardiac energy metabolism

### 3.1 Energy metabolism of the normal heart

Under normal physiological conditions, the heart generates adenosine triphosphate (ATP) through mitochondrial oxidative phosphorylation, utilizing fatty acids oxidation (FAO), glucose, lactic acid, ketone bodies, and amino acids (AA) (34, 35). This process is crucial for meeting the heart’s energy requirements. Among these energy sources, FAO is the main contributor, providing 50-70% of the ATP for muscle contraction (34, 36, 37). In normal cardiac metabolism, the heart favors fatty acids for ATP production because they are much more efficient than glucose (38). For instance, the complete oxidation of 1 mole of a 20-carbon fatty acid generates approximately 134 moles of ATP, while 1 mole of glucose only yields about 30 moles of ATP (39). Although fatty acid oxidation demands more oxygen, under aerobic conditions, its ATP-generating efficiency is considerably higher (40). Therefore, fatty acids are the preferred substrate for ATP production in a healthy heart. Glucose also plays an essential role. In anaerobic conditions, glucose undergoes glycolysis to form lactate, generating 2 ATP per molecule. In aerobic conditions, 94-97% of pyruvate enters the mitochondria for the tricarboxylic acid (TCA) cycle, and only 3-6% is converted into lactate (41). Additionally, lactic acid contributes to cardiac energy metabolism. During fasting, it can account for up to 2.8% of the ATP production in the human heart (42, 43). Recent research has revealed that under specific circumstances, lactic acid can even become the dominant supplier of pyruvate for the heart, highlighting its importance (44). Recent studies have shown that under certain conditions, lactic acid can even be the primary source of pyruvate for the heart, emphasizing its significance (45–47). Finally, amino acid oxidation, particularly of branched-chain amino acids (BCAAs), is a minor source of ATP, contributing less than 2% (34, 48, 49). This adaptability allows the heart to regulate the utilization of different energy substrates according to its needs, maintaining normal cardiac function and ensuring a continuous supply of ATP (34, 50).

### 3.2 Abnormal cardiac energy metabolism

In the state of diabetes, the heart’s metabolic processes experience substantial alterations because of a changed metabolic environment marked by hyperglycemia, hyperlipidemia, and insulin resistance (51). FAO becomes less efficient in terms of energy production and causes lipotoxicity (52). This leads to the build-up of lipid intermediates such as long-chain acyl-CoAs, acylcarnitines, ceramides, diacylglycerols, and triacylglycerols within cardiomyocytes (52). These intermediate substances interfere with mitochondrial function, cause oxidative stress, and initiate apoptosis. Moreover, insulin resistance impairs glucose uptake and utilization, further disrupting the heart’s energy metabolism (53). This metabolic imbalance makes the inefficiencies related to fatty acid oxidation even worse and contributes to overall metabolic disorder in the hearts of diabetic patients (54). Collectively, these factors result in a decrease in cardiac efficiency and an increase in oxidative stress. Eventually, they promote the development of heart failure in diabetic individuals.

In HF, the heart loses its metabolic adaptability, which throws energy metabolism into disarray. It has difficulty generating sufficient ATP, much like an engine running out of fuel (55, 56). The heart’s ability to alternate among fatty acids, glucose, and lactate as energy sources is compromised, unable to meet the high-energy requirements. The most prominent metabolic alterations in HF are a reduction in the utilization of FAO and ATP production (57–59). The capacity for fatty acid oxidation declines. Firstly, as heart failure advances, the myocardium’s capability to oxidize fatty acids diminishes (60). Secondly, the genes that code for key proteins involved in fatty acid oxidation and their regulatory factors are inhibitedOn one hand, as heart failure progresses, the myocardium’s ability to oxidize fatty acids weakens (34). When the heart switches from depending mainly on FAO to using more glucose and ketone bodies, it might further damage the myocardium.

In HF, when mitochondrial oxidative metabolism and ATP synthesis decrease, it is frequently offset by an augmented glycolytic response (61). During this compensatory process, the expression of the GLUT1 glucose transporter protein, which is a glycolytic intermediate, is upregulated (62). Simultaneously, the activity of phosphofructokinase-1 (PFK-1) rises, and the overall glycolytic flux also increases (62). However, the relatively small energy increment from glycolysis is not enough to completely counteract the cardiac dysfunction caused by energy deficiency (62). This situation might be regulated by the overexpression of mitochondrial ATPase inhibitor 1 (ATPIF1) (34, 63). It is worth noting that in cases where HF occurs concurrently with diabetes, glucose oxidation does not show an upward trend. Instead, there is an increase in anaerobic glycolysis, while aerobic glycolysis decreases (56).

Mitochondrial malfunction plays a crucial role in cardiac metabolic remodeling. It is characterized by elevated oxidative stress, disturbed calcium balance, abnormal mitochondrial dynamics, and irregular mitophagy (34). High reactive oxygen species (ROS) in cardiomyocytes cause lipid peroxidation, mitochondrial DNA damage, antioxidant depletion, and less ATP production (64). Disrupted calcium homeostasis impairs metabolic enzyme activity and activates cell-death pathways (65). Altered mitochondrial dynamics with more fission and less fusion lead to fragmented networks and lower metabolic efficiency. Dysregulated mitophagy accumulates damaged mitochondria (66).

**Figure 1:**
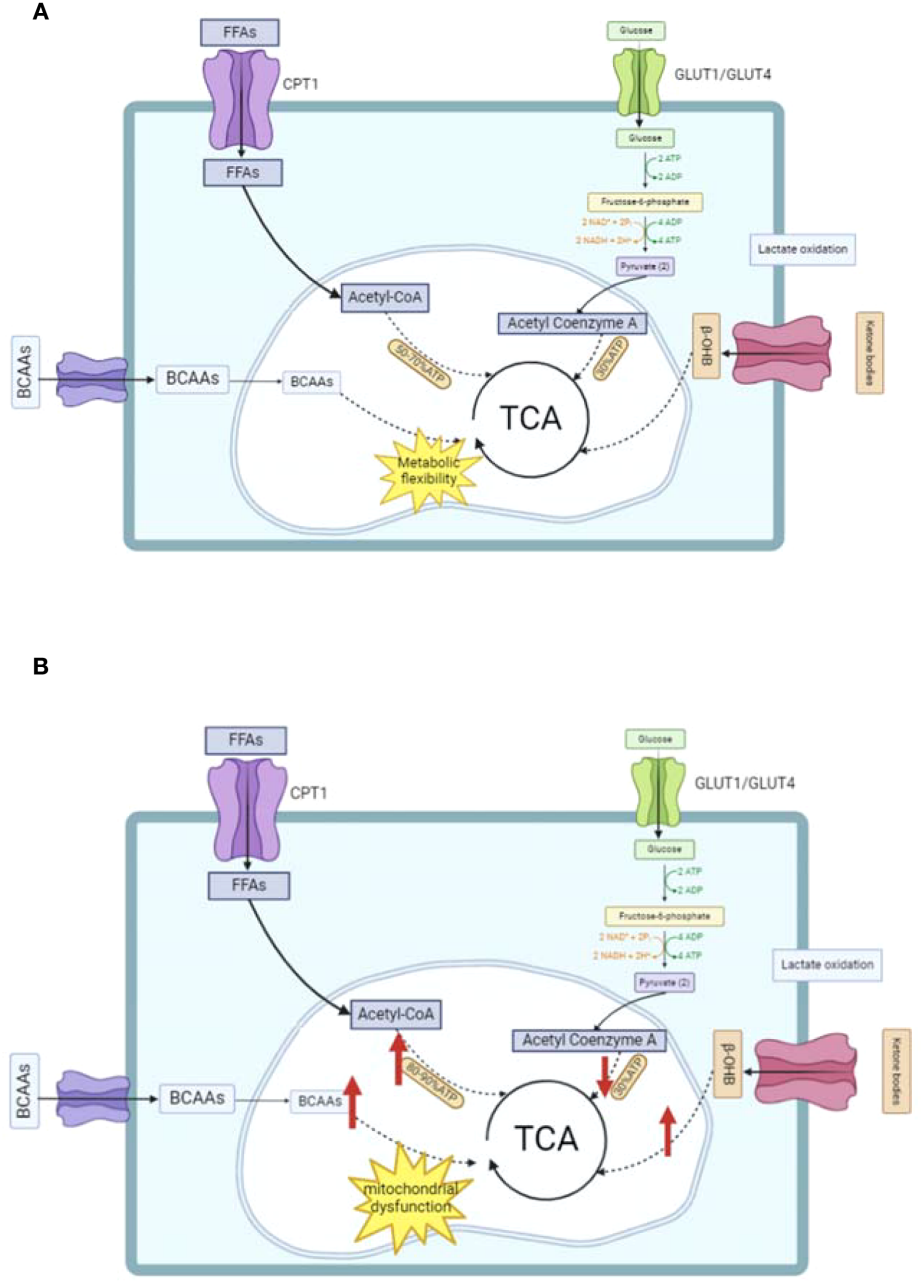
Schematic of energy metabolism in normal and abnormal hearts. **(A)** Normal cardiac energy metabolism: Illustrates the primary energy substrates (free fatty acids [FFAs], glucose, branched-chain amino acids [BCAAs]) being transported via proteins (e.g., CPT1, GLUT1/GLUT4) and integrated into the tricarboxylic acid (TCA) cycle for ATP production. **(B)** Abnormal cardiac energy metabolism: Highlights impaired fatty acid oxidation, disrupted mitochondrial function, and altered substrate utilization (e.g., increased anaerobic glycolysis) observed in conditions like diabetes or heart failure. TCA, tricarboxylic acid cycle; BCAAs, branched-chain amino acids; FFAs, free fatty acids.

When fuel metabolism is disrupted and physiological stress occurs, alternative energy sources like ketone bodies can become essential for meeting the heart’s energy demands (67). Nevertheless, high levels of ketone bodies have been associated with an increased mortality risk (68). In metabolic disorders, BCAAs often exhibit elevated concentrations (69). The buildup of BCAAs may lead to cardiac enlargement and contribute to the progression of hypertension and coronary heart disease (70, 71). Human epidemiological research has mainly shown an association between higher plasma BCAA levels in HF and unfavorable outcomes (72). The energy metabolism of normal and abnormal hearts is illustrated in **Figure 1**. The flexible utilization of energy substrates by the normal heart is central to maintaining cardiac function. However, pathological conditions such as diabetes mellitus disrupt this balance. In contrast, GLP-1RAs can regulate the aforementioned key metabolic processes to help impaired hearts restore an energy metabolism pattern approaching normality, with the specific regulatory mechanisms to be elaborated in the section on the mechanism of action of GLP-1RAs.

## 4 Mechanism of action of GLP-1RAs especially the metabolic reprogramming perspective

GLP-1RAs are hormones secreted by intestinal L cells in the ileum and colon following nutrient intake (73). Their synthesis occurs through the proteolytic processing of the proglucagon precursor by various prohormone convertases (74). These agents play a critical role in regulating postprandial glucose levels by enhancing glucose-dependent insulin secretion, a mechanism that ensures precise control of blood sugar following meals.

Accumulating clinical evidence indicates that GLP-1RAs mediate their cardioprotective actions largely via metabolic reprogramming (75, 76). This mechanism involves the modulation of fatty acid, glucose, and ketone body metabolism, mitochondrial function, as well as anti-inflammatory and antioxidant processes (77–80). Elevated plasma fatty acid levels, being associated with an increased risk of HF, can give rise to lipotoxicity (81). Such lipotoxicity induces cardiotoxic effects through bioactive sphingolipids like ceramides and diacylglycerols (DAGs) (82). GLP-1RAs mitigate these adverse impacts by enhancing fatty acid oxidation via the Creb5/NR4a1 signaling axis, thereby reducing mitochondrial damage, lipid accumulation, and ATP deficiency (77, 78, 83). This metabolic regulation diminishes lipotoxic stress and optimizes cardiac energy utilization, conferring direct cardioprotective benefits (83). Additionally, GLP-1RAs reduce levels of cholesterol, low-density lipoprotein (LDL), and triglycerides, thereby decreasing the likelihood of cardiovascular events (84–86). Preclinical investigations have shown that these agents downregulate proprotein convertase subtilisin/kexin type 9 (PCSK9) expression, upregulate low-density lipoprotein receptor (LDLR) levels, and suppress postprandial secretion of triglycerides and chylomicrons (87, 88). For example, exendin-4 lowers very-low-density lipoprotein cholesterol (VLDL-C) and LDL-C in animal models by reducing hepatic sterol regulatory element-binding protein 2 (SREBP2) levels and cholesterol absorption (88). Tapolutide has been shown to decrease total cholesterol, LDL-C, triglycerides, and hepatic steatosis (84). Tirzepatide further attenuates lipopolysaccharide (LPS)-induced left ventricular remodeling and dysfunction by inhibiting the TLR4/NF-κB/NLRP3 inflammatory pathway (89). Collectively, these actions improve lipid profiles and alleviate lipotoxic burdens on the myocardium, contributing to the comprehensive cardioprotective effects of GLP-1RAs.

GLP-1RAs improve glucose uptake in cardiomyocytes through dual mechanisms: triggering AMPK activation to facilitate GLUT4 translocation to the cell membrane and regulating the insulin signaling pathway to upregulate GLUT4 expression (79, 80). These agents further optimize glucose utilization by activating glycolytic enzymes such as hexokinase and phosphofructokinase, thereby enhancing glycolytic flux (90). GLP-1RAs also alleviate high-sugar-induced dysfunction in endothelial progenitor cells through the SDF-1β/CXCR7-AMPK/p38-MAPK/IL-6 signaling axis (91). During ischemia or periods of high energy demand, GLP-1RAs enhance ketone body utilization, providing additional energy for cardiomyocytes (92). This process is vital for reducing oxidative stress and damage during myocardial ischemia-reperfusion injury. In terms of mitochondrial function, GLP-1RAs act through multiple pathways: first, stimulating mitochondrial biogenesis via the AMPK signaling pathway to boost both the number and functionality of mitochondria (93). Second, regulating mitochondrial dynamics to decrease fragmentation and optimize morphological and functional integrity (94). Finally, GLP-1RAs decrease ROS to ease oxidative stress and shield mitochondria (94). Their anti-inflammatory and antioxidant properties further contribute to mitigating cardiac injury and improving overall cardiovascular health. Proteomic studies in T2DM patients show that liraglutide treatment enhances cardiac-metabolic profiles by modulating 72 key proteins involved in acute-phase responses, chronic inflammation, and oxidative stress-changes that may improve heart health outcomes (95). Additionally, GLP-1RAs therapy increases circulating vascular progenitor cell content while reducing proinflammatory granulocyte precursor levels, representing an additional mechanism underlying their cardioprotective effects (96).

**Figure 2:**
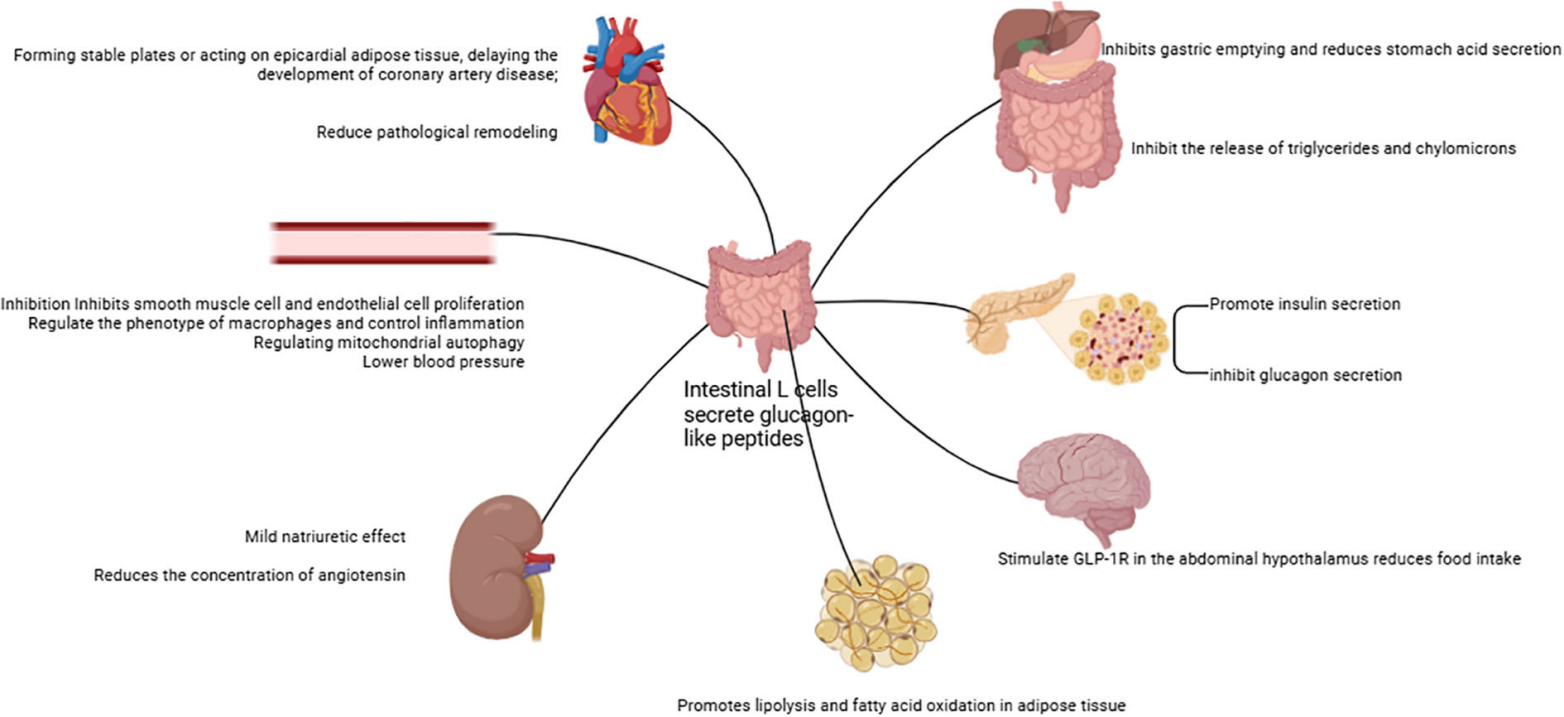
The GLP-1R distribution.

The glucagon-like peptide-1 receptor (GLP-1R) is ubiquitously present across multiple bodily tissues, including the pancreas, lungs, kidneys, central nervous system, cardiovascular system, gastrointestinal tract, as well as skin and vagus nerves (its tissue distribution is illustrated in **Figure 2**) (97). By binding to these receptors, GLP-1RAs induce calorie expenditure through mechanisms that mimic a fasting-mimicking metabolic state. This adaptive pattern triggers systemic adjustments in energy metabolism, encompassing glucose homeostasis, hormonal secretion, energy substrate utilization, and energy expenditure regulation.

**Figure 3:**
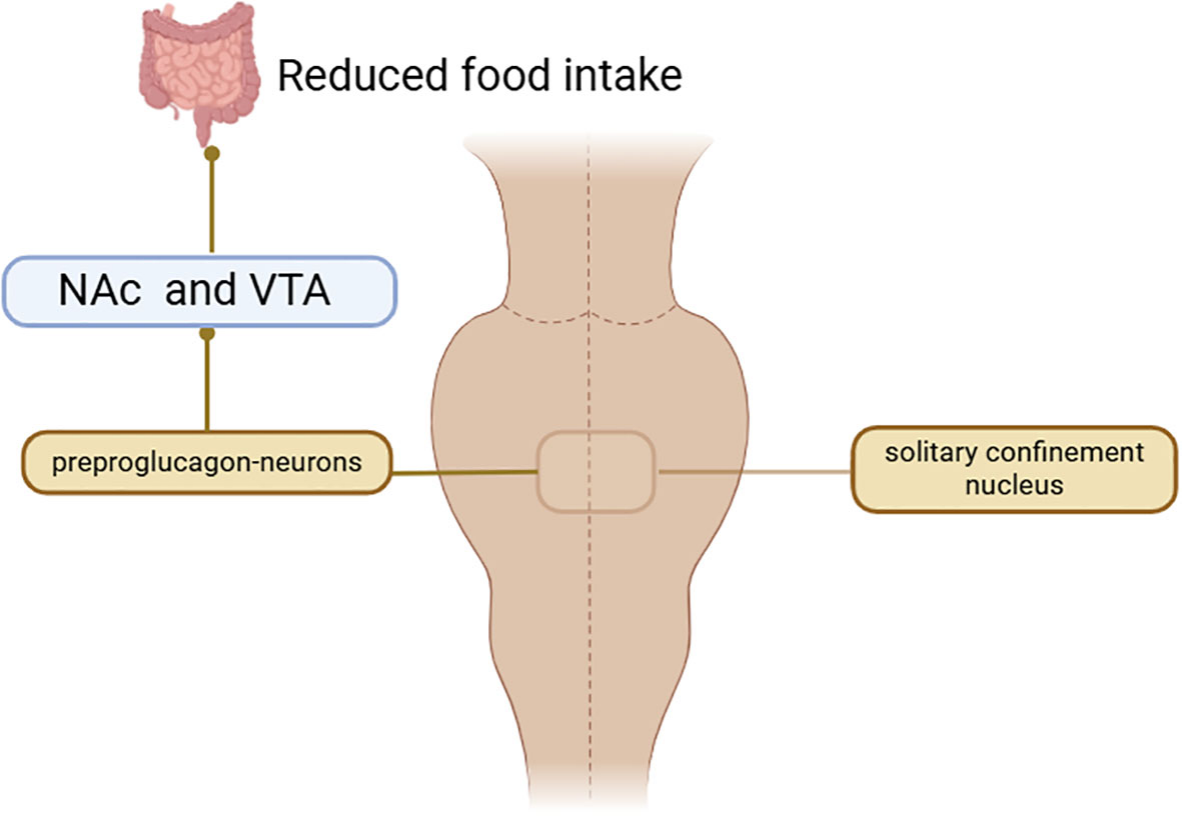
GLP-1RAs main mechanism for inhibiting food intake.

**Figure 4:**
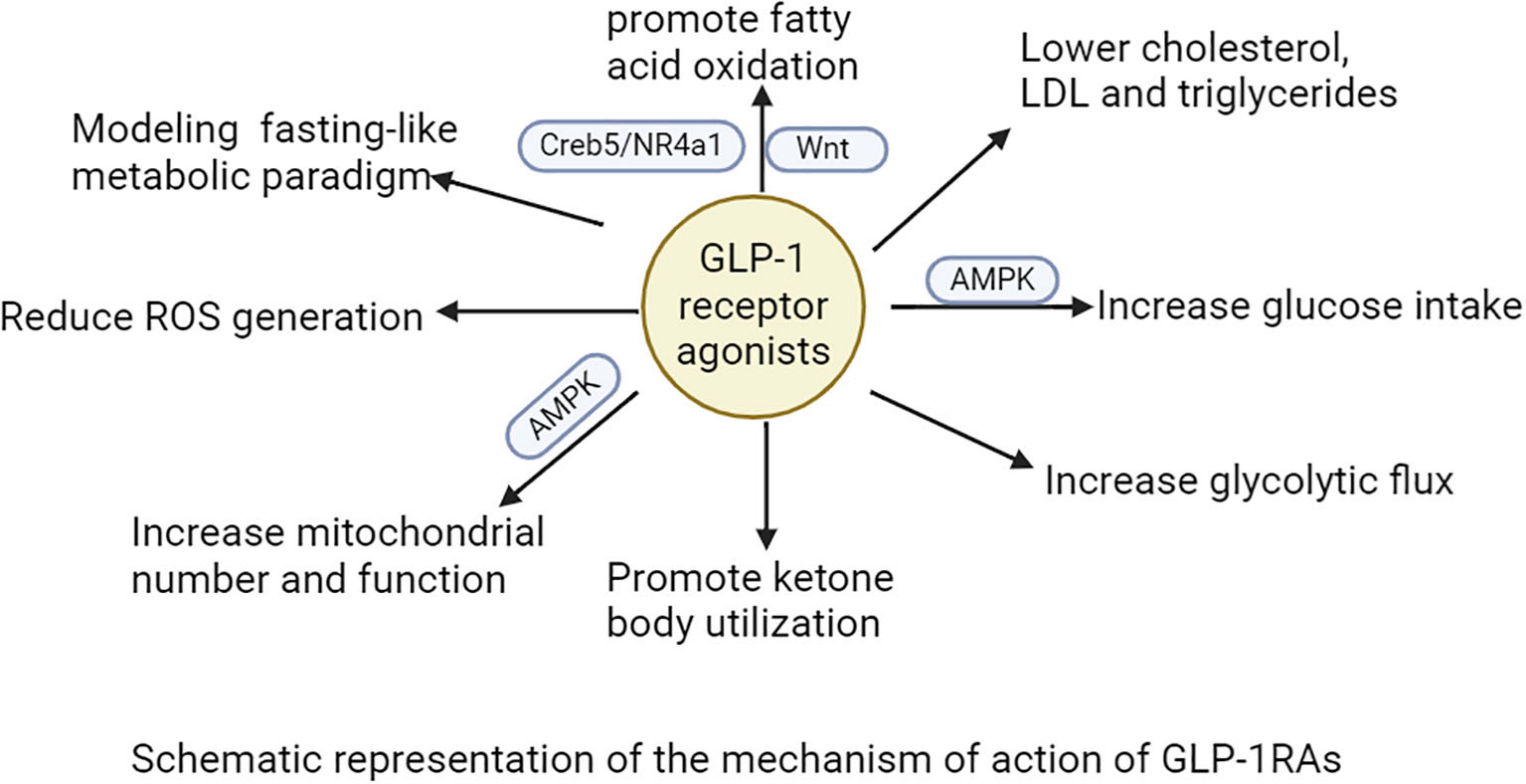
The mechanism of action of GLP-1RAs is illustrated.

**Figure 5:**
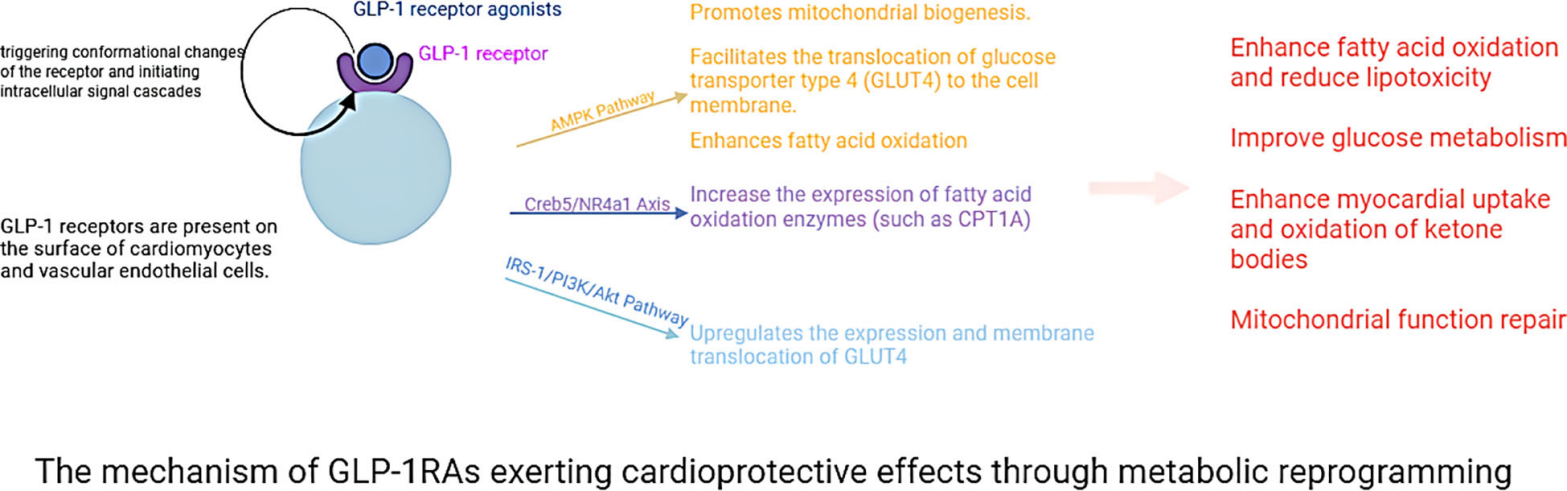
The mechanism of GLP-1RAs exerting cardioprotective effects through metabolic reprogramming.

Within the central nervous system, GLP-1RAs function to reduce hunger sensations, suppress appetite, lower caloric intake, amplify satiety, and facilitate better management of eating behaviors (98–102). Activation of GLP-1R in the hypothalamic paraventricular nucleus (PVN) triggers an appetite-suppressing response through neural pathways involving corticotropin-releasing hormone (CRH) excitatory neurons (103, 104). Preclinical studies have indicated that GLP-1RAs require AMPK inhibition to exert their anorectic effects (105). AMPK is a nutrient and glucose sensor in the hypothalamus that is affected by substances such as blood glucose、intracellular energy levels, leptin、GHrelin releasing peptide, and MT-2136 (106–109). This regulatory mechanism is central to how GLP-1RAs control energy intake, as outlined in the primary pathways for food intake inhibition shown in **Figure 3**. Beyond central nervous system actions, the weight-loss effects of GLP-1 analogs also involve peripheral metabolic adaptations. These agonists can facilitate the transformation of visceral white adipose tissue (WAT) into brown adipose tissue (BAT), thereby stimulating BAT thermogenesis through sympathetic nervous system activation to enhance energy expenditure (110–112). The comprehensive mechanism of action for GLP-1RAs is depicted in **Figure 4**. And GLP-1RAs exert cardioprotective effects through metabolic reprogramming is illustrated in **Figure 5**.

## 5 Conclusion

Diabetes mellitus imposes a heavy burden on public health worldwide, particularly due to its close association with increased incidence and mortality of cardiovascular diseases. Clinical evidence consistently demonstrates that GLP-1RAs exert cardioprotective effects, including a significant reduction in the risk of MACE, alleviation of heart failure-related symptoms, and delay in the progression of atherosclerotic lesions. For instance, semaglutide alleviates physical limitations in HFpEF patients, while tirzepatide reduces the composite endpoint of cardiovascular death or worsening HF in obese patients with HFpEF-underscoring their broad utility in cardiometabolic disease management. The notable cardioprotective benefits  of GLP-1RAs have spurred exploration into their mechanistic actions beyond glycemic control. Recent investigations into their broad influences on glucose, lipid, and protein metabolism have provided fresh insights into deciphering the advantageous cardioprotective effects of this drug class in T2DM-related cardiovascular disorders. Intriguingly, GLP-1RAs initiate systemic metabolic reprogramming that emulates a fasting-like state to regulate metabolic processes and energy balance. This reprogramming entails enhanced glucose utilization, optimized lipid metabolic pathways, and improved protein homeostasis-all of which likely contribute to their cardiorenal protective actions. Specifically, GLP-1RAs boost fatty acid oxidation, facilitate glucose uptake and utilization, enhance mitochondrial function, and increase ketone body utilization. Collectively, these mechanisms optimize cardiac energy metabolism, mitigate oxidative stress, and support overall cardioprotection. Further research is needed to resolve uncertainties regarding the specific metabolic alterations and to achieve a comprehensive understanding of how GLP-1RAs affect metabolism and the underlying molecular pathways. Future studies should assess whether the metabolic reprogramming induced by GLP-1RAs exhibits dose-dependent characteristics and employ bibliometric approaches to investigate the temporal patterns of these metabolic changes.
